# The contribution of Ghanaian pharmacists to mental healthcare: current practice and barriers

**DOI:** 10.1186/1752-4458-4-14

**Published:** 2010-06-15

**Authors:** Frances T Owusu-Daaku, Afia FA Marfo, Edmund A Boateng

**Affiliations:** 1 Department of Clinical & Social Pharmacy, Kwame Nkrumah University of Science and Technology, Kumasi, Ghana

## Abstract

**Background:**

There is scant knowledge of the involvement of developing country pharmacists in mental healthcare. The objectives of this study were: to examine the existing role of Ghanaian community and hospital pharmacists in the management of mental illness, and to determine the barriers that hinder pharmacists' involvement in mental healthcare in Ghana.

**Method:**

A respondent self-completion questionnaire was randomly distributed to 120 superintendent community pharmacists out of an estimated 240 pharmacists in Kumasi, Ashanti Region of Ghana. A purposive sampling method was utilized in selecting two public psychiatric hospital pharmacists in Accra, the capital city of Ghana for a face-to-face interview. A semi-structured interview guide was employed.

**Results:**

A 91.7% response rate was obtained for the community pharmacists' questionnaire survey. Approximately 65% of community pharmacists were not involved in mental health provision. Of the 35% who were, 57% counseled psychiatric patients and 44% of these dispensed medicines for mental illness. Perceived barriers that hindered community pharmacists' involvement in the management of mental health included inadequate education in mental health (cited by 81% of respondents) and a low level of encounter with patients (72%). The psychiatric hospital pharmacists were mostly involved in the dispensing of medicines from the hospital pharmacy.

**Conclusion:**

Both community and hospital pharmacists in Ghana were marginally involved in the provision of mental healthcare. The greatest barrier cited was inadequate knowledge in mental health.

## Background

Mental and behavioral disorders are estimated to account for 12% of the global burden of disease. The World Health Organization (WHO) estimates that 25% of any population will suffer from neuro-psychiatric conditions during their lifetime [1]. In Ghana there is no current published data on the prevalence of mental illness. However, 77,688 and 82,819 patients attended the three major mental health institutions in 2001 and 2002 respectively [2]. The commonest case diagnosed at these institutions was schizophrenia.

On 9^th ^October 2008 in Geneva, WHO launched its mental health gap action program (mhGAP). This aims at scaling up services for mental, neurological and substance use disorders, particularly in countries with low and middle incomes. WHO believes that with proper care, psychosocial assistance and medication, tens of millions could be treated for depression, schizophrenia, and epilepsy, prevented from suicide and begin to lead normal lives even in resource-limited settings [3].

Health professionals have identified people with mental illness as among their most challenging patients to manage [4]. Appropriate drug therapy helps treat mental health problems, minimize relapse and reduce suicide [5]. Consequently, the primary goal of the pharmacist in mental health provision should be the safety and well-being of the patient, ensuring that patients who need medicines receive them and adhere to treatment [6]. Indeed, WHO has acknowledged pharmacists as dynamic members of the mental healthcare team who should assist in improving psychotropic medication use [7].

In developed countries, pharmacists are involved in both in-patient and out-patient psychiatric settings. Pharmacists routinely participate as pharmacotherapy experts and consultants, manage individual patient drug regimens, obtain drug histories, lead patient education discussion groups, and coordinate the use of drug assistance programs. A retrospective chart review has indicated that duties performed by pharmacists in psychiatric settings can improve physician prescribing practices and enhance treatment outcomes [8]. These positive outcomes, associated with savings in drug acquisition costs could, theoretically, reduce the potential side effect burden of the psychotropic agents administered and could reduce the numbers of potentially inappropriate psychotropic medications prescribed for mental illness [9-11]. Pharmacists in the United Kingdom play a role at every stage of the patient's recovery, whether patients are being cared for in an acute setting or in the community. Pharmacists assess and advise on medication and are also involved in lithium and depot injection clinics at the hospital and in the community [12].

In a developing West African country like Ghana, little is known about the contribution of pharmacists to mental healthcare. This study seeks to provide current and relevant information for the purposes of policy initiation, planning and decision making, especially since the Ghana mental health act is being reviewed. Healthcare professionals, notably practicing pharmacists, will also be informed on the current as well as the possible role the pharmacist could play in mental health delivery.

The objectives of the study were to examine the existing role of the Ghanaian community and hospital pharmacist in the management of mental illness and to determine the barriers that hinder pharmacists' involvement in the management of mental healthcare.

## Method

### Study sample

The study covered community pharmacists in the city of Kumasi and the two psychiatric hospitals in Accra (Pantang hospital and Accra psychiatric hospital). These areas were selected because Pantang hospital and Accra psychiatric hospital have the largest number of mental patients in Ghana; and the city of Kumasi, a convenient setting for the researchers, is the second largest city in Ghana with approximately 240 community pharmacies.

In phase one of the study, a simple random sampling technique was used to select 120 community pharmacies out of the estimated 240 community pharmacies in Kumasi. In phase two, purposive sampling was used to select the pharmacist in charge of each of the two psychiatric hospitals for an interview. These pharmacists were assumed capable of providing extensive information on pharmacy practice at the hospital, having worked for at least five years in that position. At the time of the study, there were only two pharmacists employed in each of these hospitals.

### Development and validation of the survey instrument

The respondent self-completion questionnaire and the semi-structured interview guide were developed primarily by the researchers based on literature regarding pharmaceutical care offered by community and hospital pharmacists, with emphasis on mentally-challenged patients. The questionnaire for community pharmacists was aimed at determining pharmacists' role and knowledge of, barriers to their increased involvement in, and their views on regular workshops for Ghanaian community pharmacists on the management of mental illness (Appendix 1). The semi-structured interview guide, designed for a face-to-face interview with the hospital pharmacist, involved discussions on the current role of the pharmacist on admission, ward rounds, discharge, and views on the formation of medication education groups among mentally-challenged patients (Appendix 2). Both of these instruments were then reviewed by a fellow researcher, a practicing community pharmacist and a hospital pharmacist. They were also pre-tested on a sample of five community pharmacists and a hospital pharmacist respectively, to check for precision, ease of understanding and flow of content.

### Ethical considerations

Permission for the study was sought from both the Pharmaceutical Society of Ghana and the respective hospital directors. Pharmacists were then informed about the study and their consent sought before the questionnaire was administered.

### Data collection

In phase one of the study, the 120 community pharmacies chosen for the study were visited from the 1^st ^to 5^th ^February 2009. The pharmacists in the selected community pharmacies were then given two weeks to complete the questionnaire and these were retrieved from the respondents by EAB.

In phase two, appointments were booked for a face-to-face interview with the pharmacists in charge of the hospitals. The first appointment was on the 8^th ^of January 2009 and the second appointment was on the 13^th ^of January 2009. Both interviews were conducted by EAB.

### Data analysis

Qualitative data was grouped into themes and SPSS version 15 was used to obtain descriptive analysis of the data collected.

## Results

### Response rate

Of the 120 questionnaires administered to the community pharmacists, 110 were retrieved representing a response rate of 91.7%.

### Demographic characteristics of community pharmacists

25% of the respondents were females and 75% were males. The number of years of practice ranged from 1-5 years (50%), 6-10 years (41%) and over 10 years (9%).

### Existing role of community pharmacists

65% of respondents did not play any role in the management of mental health illness. However with regard to the 35% who did, the highest proportion (57%) was involved with counseling associated with dispensing of medicines to psychiatric patients (Table 1).

**Table 1:** Roles of community pharmacists in the management of mental illness

Roles	Frequency (Percentage)
Counseling	22 (57)
Dispensing of drugs	17 (44)
Provision of drug information	12(32)
Patient monitoring	8 (21)

### Community pharmacists' knowledge of psychotropic medicines

Seventy nine percent (79%) of respondents rated their knowledge on psychotropic medicines as average, 2% as good and 19% as poor. The main reason cited for a less than good rating was inadequate education in the area of mental health (Table 2).

**Table 2:** Reasons cited by community pharmacists for rating knowledge of psychotropic medicines as average or poor

Reason	Frequency (Percentage)
Inadequate education in this area	80 (73)
Few prescriptions for mental health drugs	73 (66)
Inadequate material on mental health therapy	57 (45)

### Views of community pharmacists on regular workshops

All the respondents said regular workshops would help improve their involvement in mental healthcare delivery. Workshops could cover signs and symptoms of mental illness (65% of respondents), counseling (96%), psychotropic medicines (79%) and current treatment regimens for mental illness (86%).

### Barriers hindering community pharmacists' involvement

Perceived barriers that hindered the community pharmacist's involvement in the management of mental health included inadequate knowledge in the area (cited by 81% of respondents), low level of encounter with mental patients (72%), poor remuneration (56%), stigmatization of patients and personnel in the field (59%) and inadequate funding of services (51%).

### Role of hospital pharmacists' on patient admission

The two respondents said they played a role on patient admission:

"*On admission we check for the appropriateness of any prescribed drugs, relating to the dose- and interaction with other medication when the prescription is brought to the pharmacy*." (H1)

"*On admission, we dispense any drugs needed and help in stabilizing agitated patients by providing sedatives*." (H2)

However, there was hardly any encounter between pharmacists and patients, or pharmacists and carers during admission; for medicine use review, counseling and monitoring of therapy:

"... *admission is mostly done at the records department so we do not get to meet the carers. Also, most often, the nurses come here for all the patients' medications."*(H1)

*"We do not do much drug history taking or review here..." *(H2)

Although the pharmacists did not see the carers or patients on admission, they were of the opinion that it was important for them to do so:

"...*it could afford us the opportunity to counsel carers on the medication of the patient and it could also help us to know our patients better*." (H1)

"*It could help to foster good relations between the pharmacist and the carers of the patients..."*(H2)

### Role of hospital pharmacists during ward rounds

Respondents reported that they seldom conducted ward rounds and when they did, they did not go with other members of the healthcare team. One respondent cited his inadequate knowledge in psychiatry as a reason why he did not go on ward rounds with other members of the healthcare team:

"*Well, on my part, I do not normally go for ward rounds due to my limited knowledge in psychiatry. You know in school we are not taught much of psychiatric conditions and this would make contributions during ward rounds difficult."*(H1)

The other respondent explained that when she joined the hospital there was no structured ward rounds with other members of the healthcare team,

"... *that is what is done here. I came to meet that here." *(H2)

### Role of hospital pharmacists during discharge of patients

Both respondents reported that they did not play a meaningful role during the discharge of patients.

"*When a patient is discharged, usually the nurses come here for their drugs instead of their carers. So we counsel the nurses on how the patients should take their medication and hope they pass it on to the carers or the patients themselves." *(H1)

"*Ideally, we should have followed up on patients after discharge but this is not done here because there are community mental health nurses who provide primary care to discharged patients at the community level. However, we sometimes help in determining the time a discharged patient should come for review." *(H2)

### Hospital pharmacists' views on formation of medication education groups

It was apparent from the interviews that the respondents had mixed feelings about the formation of such groups, though they generally endorsed the view that if well-formed, the groups could help improve drug compliance.

"*Yes because if properly done, it could help to educate patients on their medication, especially on issues of drug compliance by letting them know why they take their drugs. On the other hand, I would say no, because most of these patients have communication problems. Some do not know what they are talking about and others even do not understand what is said to them. Educating such a group will be very difficult if not impossible." *(H1)

"*No, because most of the patients we have here are not literate. Most of them even attach superstitious beliefs to their illness. This will make communicating with them very difficult and I wonder how it could help in their drug therapy since most do not believe in the drugs anyway." *(H2)

## Discussion

### Community pharmacists' involvement

Of the 35% community pharmacists who reported playing a role in the management of mental illness, only 44% of these were involved with the dispensing of medicines to psychiatric patients (Table 1). This is not surprising because in Ghana most psychotropic medicines are dispensed free of charge to patients at the government psychiatric hospitals, thus patients visit the community pharmacies to purchase medicines when the psychiatric hospitals are out of stock or do not stock the psychotropic medicines prescribed.

### Community pharmacists' perceived knowledge and challenges

Ninety eight percent (98%) of respondents rated their knowledge in psychotropic medicines as either average or poor. This perceived inadequate education is likely to be an important factor limiting their involvement in the management of mental illness (the reason given by 81% of the respondents for their non-involvement). This corroborates with a study conducted in Belgium which showed that lack of training in mental health issues was the most important barrier hindering pharmacists' role in depression [13]. Although pharmacists are generally experts in drug therapy, offering efficient pharmaceutical care to mentally challenged patients can be quite daunting. Studies have indicated that the provision of information about medications by community pharmacists may be limited by poor communication with people with mental illnesses [14]. Pharmacists have reported feeling more uncomfortable counseling on the use of medications used to treat mental illnesses than those used to treat cardiovascular conditions [15]. Hence specific knowledge and skills are required so as to optimise treatments for mentally challenged patients.

All respondents were of the view that regular workshops would help improve their knowledge in mental health care delivery. Pharmaceutical governing bodies, such as the Pharmacy Council of Ghana, could help by organizing these workshops for community pharmacists and psychiatric hospital pharmacists to ensure that they are equipped to perform their expected roles. Topics to be covered could include; communication with mentally challenged patients, pharmacotherapy and other duties expected of the pharmacist as proposed by the community pharmacists. However to adequately organise these workshops, pharmacists and resource persons who are experts in mental health would be needed. Currently Ghana has nine consultant psychiatrists and no specialist psychiatric pharmacist [Personal Communication, office of the Chief Psychiatrist of Ghana]. Hence healthcare personnel, especially psychiatric pharmacists in the developed countries, could assist by volunteering their services so as to upgrade psychiatric pharmacy in Ghana. Furthermore universities offering pharmacy in Ghana could offer postgraduate courses on psychiatric pharmacy. In the United States there are approximately 25 to 30 one-year, post-Pharm. D residencies in psychiatric pharmacy practice, with 17 receiving accreditation by the American Society of Health-System Pharmacists (ASHP) [16,17]. Each residency may offer a unique feature such as an ambulatory care focus or teaching skills development; however, the emphasis of residency training is on specialized clinical knowledge and skills development.

Seventy nine percent (79%) of the community pharmacists who responded to the questionnaire had never been consulted by discharged mental patients or their carers for advice on their medication. This could mean one of two things: either mental illness was not a common condition in the community or most people with the condition did not perceive the pharmacy as a place where they could go for consultation on their medication.

Records as at 2002 showed that 4,972 mental patients were discharged from the Ankaful, Pantang and Accra psychiatric hospitals (Ankaful is the 3rd major psychiatric hospital in Ghana) [2] and in a newspaper report on 8^th ^of August, 2008, the acting Chief Psychiatrist of the Accra psychiatric hospital stated that about 440,000 Ghanaians suffered a severe form of mental illness[18]. The second reason, that most people with mental conditions do not perceive the pharmacy as a place where they could go for consultation on their mental health medication, is therefore more plausible and hence it is essential that the mental health law that is being reviewed includes community pharmacists as part of the community mental health teams. This would also address the problem of low encounter with patients cited by 72% as a perceived barrier for hindering their involvement in mental health care delivery. Duties of community psychiatric nurses include awareness creation and mental health promotion, identification of cases and referral of cases to specialist hospitals and management of some cases including counseling [2]. These duties and more could be performed by community pharmacists working together with community psychiatric nurses to upgrade pharmaceutical care of mentally challenged patients. There is evidence that suggests that pharmacists have a potential role as members of community mental health teams. Pharmacists' participation in community psychiatry clinical team meetings created an opportunity to present medication review findings and recommendations^. ^Pharmacists were also perceived as a valuable source of unbiased and evidence-based drug information for both mental health team staff and their clients and caregivers [19]. There is also evidence to the effect that counseling provided by community pharmacists to discharged mental patients significantly contribute to improved medication compliance [4]. However adequate remuneration should be given through the National Health Insurance Scheme (NHIS) to community pharmacists to enhance their interest in this area since poor remuneration was cited by 56% as a perceived barrier that hinders their involvement in mental healthcare. Community pharmacies are also more accessible to the general population, their opening hours are usually convenient and they provide personalised services [20]. Therefore their involvement will also aid in decentralization of mental healthcare, resulting in greater accessibility.

Public awareness of the role played by pharmacists in mental health delivery is essential, as this will increase the level of pharmacists' encounter with mentally-challenged patients. These services would then not seem strange to clients as perceived by pharmacists in this study. Most Ghanaians believe conditions like mental illness, convulsions and epilepsy to be spiritual and hence require spiritual attention [2]. Thus most carers of patients with such beliefs would rather take discharged patients showing signs of relapse or discomfort due to side effects of psychotropic medications to spiritual healers than seek advice from a pharmacist.

To address perceived barriers of stigmatization of patients and personnel in the field and inadequate funding as cited by 59% and 51% of community pharmacists respectively, relevant policies should be formulated in addition to other policies that avoid controversies over roles within the healthcare team; and regulate traditional healers, prayer camps and other unorthodox institutions. The mental health law of Ghana is being revised to promote the care, and to protect the human rights, of people with mental illness [21]. In 2006 the Government of Ghana gave approval to the Ministry of Health (MOH) Ghana to revise 19 outmoded health related laws in order to improve health service delivery in the country. The MOH is currently working on seven of these laws, including the Mental Health Law which was last reviewed in 1972. Mental health service decentralization, infrastructure for management and rehabilitation of the mentally ill in the community, condition of service in mental service, mental health awareness and transportation for community health services are being reviewed [2,21].

### Hospital pharmacists

The study revealed that on admission, the psychiatric hospital pharmacist checks for the appropriateness of any drug prescribed for the patient, whiles dispensing the medicines at the pharmacy. This does not differ widely from what pharmacists in the general hospital setting do. However, it was identified that on admission the pharmacist did not usually see the patient or the carer for a medication review or drug history documentation, and all information about prescribed medication was given to the patient through the nurses. This is not the best approach to optimize pharmaceutical care. Correct documentation of a patient's medication history is an important part of the initial patient assessment after admission to hospital [22]. Pharmacists, rather than physicians, have been shown to obtain more accurate medication-related information from patients [23]. Accurate medication history enables the healthcare team to have an idea of all medications, including creams and any herbal preparations the patient had taken, any of which might even be a contributing factor to the presenting illness.

Pharmacists in both hospitals did not usually conduct ward rounds with other members of the healthcare team. Lack of adequate team work between the pharmacist and other members of the healthcare team was also identified as a barrier to their involvement in ward rounds. Ward rounds have been identified as the best opportunity for pharmacists to contribute to patient care. It is where decisions are made about the patient's care and it is therefore the place where pharmacists can ensure that patients are receiving the correct treatment [12].

The pharmacists in the two hospitals did not normally see the patients when they were discharged. The nurses usually obtained the patient's drugs from the pharmacy and thus all information about the drugs was given to the patient via the nurses. It is, however, important for the pharmacists to be involved in planning the discharge care and to interact with the patient and their carers. This would help solve any issues with medications and foster a good relationship between the pharmacist, patient and carer [12].

Issues such as poor communication with patients and the fact that most of the patients and their carers attached superstitions to their condition could make the formation of medication education groups difficult. However, the formation of such groups could provide a forum for patients to discuss their therapy and ask questions on their medications. This could improve drug compliance and thus reduce the frequency of relapses.

Policies should be in place detailing the role of the hospital pharmacist in psychiatric hospitals in Ghana so as to enhance pharmaceutical care of patients admitted at the psychiatric hospitals since studies have shown that the presence of the pharmacist on the ward improves accuracy of drug history documentation, reduce prescribing cost and decrease the potential risk of patients in the hospital [24].

## Conclusion

The survey demonstrated that Ghanaian pharmacists in community practice did not play any significant role in the management of mental illness, were restricted by several barriers in involving themselves in the management of mental illness and would welcome regular workshops on mental health. The psychiatric hospital pharmacists were mostly involved in dispensing medicines from hospital pharmacies. However they seldom played a role in offering pharmaceutical care to psychiatric patients on admission or during discharge. Barriers that hindered these hospital pharmacists were similar to those perceived by the community pharmacists and it is essential that these barriers are addressed.

### Appendix 1: Current role and knowledge of community pharmacists in management of mental illness

1. Do you play any role in mental health care delivery Yes { } No { }

2. If yes, which of these duties do you perform (please tick)

a) Dispensing of psychotropic medicines

b) Counseling of patients mentally challenged

c) Patient monitoring

d) Provision of drug information (esp. psychotropic medicines)

e) Conducting medicines use reviews

3. Has any discharged mental patient or carer of a discharged mental patient ever been to you for advice on their medication? { } yes { } no

4. If yes, how would you rate their satisfaction after talking to you?

{ } excellent { } poor { } good { } very poor { } average

5. How would you rate your current knowledge on mental health drug therapy?

{ } excellent { } poor { } good { } very poor { } average

6. Please rate your interest in involving yourself in the management of mental illness

{ } excellent { } poor { } good { } very poor { } average

7. Roughly, what percentage of the drugs in your pharmacy are psychotropic agents? (0-100%)...........................................................

8. If you scored below 20% above, please explain why you have a low stock of psychotropic drugs

..................................................................................................................

.....................................................................................................

9. Please list some barriers to your increased involvement in the management of mental illness.

❖ .............................................................

❖ .............................................................

❖ .............................................................

❖ .............................................................

❖ .............................................................

10. Do you think regular workshops for community pharmacists on mental health drug therapy will improve how you handle cases of mental illness?

{ } yes { } no

11. If yes, please explain

.....................................................................................................

12. If no, please explain

.....................................................................................................

13. Which areas do you think such a workshop should cover?

❖ .............................................................

❖ .............................................................

❖ .............................................................

Personal information

14. Age...................

15. Number of years of practice

16. Sex Male/Female

### Appendix 2: Semi structured guide for face to face interview with hospital pharmacist

#### Role on patient admission

1. Briefly, what role does the hospital psychiatric pharmacist play on patient admission?

2. Are there any differences between the role played by pharmacists in the general hospital and those played by the pharmacist in the psychiatric hospital?

3. What does drug history review for newly admitted mental patients entail?

4. Does the pharmacist in the psychiatric hospital interview carers of patients on admission?

5. What do those interviews entail?

6. Do you think it is important for the hospital psychiatric pharmacist to interview carers of mental patients on admission?

#### Role played on multidisciplinary team meetings and ward rounds

1. What role does the pharmacist in the psychiatric hospital play during ward rounds?

2. In some hospitals, the pharmacist goes on ward rounds alone first, before going with the other healthcare professionals. Is the same thing done here?

3. Do you think such a practice is good?

4. Briefly, what are some of your challenges during ward rounds?

5. How do you propose such challenges could be overcome?

#### Role played during discharge of patients

1. What role do you play in planning the discharge care of patients?

2. What measures do you put in place to ensure compliance of drug therapy of discharged mental patients?

#### Views on formation of medication education groups

1. Do you think the formation of medication education groups of mental patients will help in drug therapy?	Please explain.

2. What role could the psychiatric hospital pharmacist play in such groups?

3. What are some of the barriers to the formation of such groups in your hospital?

## Competing interests

FTOD is the former Vice President of the Pharmaceutical Society of Ghana

AFAM is a member of the Pharmaceutical Society of Ghana

EAB is a member of the Ghana Pharmaceutical Students Association

## Authors' contributions

Conception, design, acquisition and analysis of data - FTOD and EAB

Drafting of manuscript and revising it - FTOD and AFAM

Revising and approval of final manuscript to be published: FTOD, AFAM and EAB

## Authors' information

1.FTOD

Qualifications: PhD (Manc) MSc and B. Pharm (Ghana)

Current positions held at institutions or societies: Senior Lecturer and Head, Department of Clinical and Social Pharmacy, Kwame Nkrumah University of Science and Technology, Ghana

2. AFAM

Qualifications: B. Pharm (Ghana); MSc. Clinical Pharmacy, International Practice & Policy (ULSOP, London)

Current positions held at institutions or societies: Lecturer Department of Clinical & Social Pharmacy, Kwame Nkrumah University of Science and Technology, Ghana

3. EAB

Qualifications: B.Pharm (Ghana)

Current positions held at institutions or societies: Pharmacist Intern
